# Associations of arterial carbon dioxide and arterial oxygen concentrations with hospital mortality after resuscitation from cardiac arrest

**DOI:** 10.1186/s13054-015-1067-6

**Published:** 2015-09-29

**Authors:** Hendrik J. F. Helmerhorst, Marie-José Roos-Blom, David J. van Westerloo, Ameen Abu-Hanna, Nicolette F. de Keizer, Evert de Jonge

**Affiliations:** Department of Intensive Care Medicine, Leiden University Medical Center, Post Box 9600, Leiden, 2300 RC The Netherlands; Laboratory of Experimental Intensive Care and Anesthesiology, Academic Medical Center, Meibergdreef 9, Amsterdam, 1105 AZ The Netherlands; Department of Medical Informatics, Academic Medical Center, Meibergdreef 9, Amsterdam, 1105 AZ The Netherlands; National Intensive Care Evaluation (NICE) foundation, Meibergdreef 9, Amsterdam, 1105 AZ The Netherlands

## Abstract

**Introduction:**

Arterial concentrations of carbon dioxide (PaCO_2_) and oxygen (PaO_2_) during admission to the intensive care unit (ICU) may substantially affect organ perfusion and outcome after cardiac arrest. Our aim was to investigate the independent and synergistic effects of both parameters on hospital mortality.

**Methods:**

This was a cohort study using data from mechanically ventilated cardiac arrest patients in the Dutch National Intensive Care Evaluation (NICE) registry between 2007 and 2012. PaCO_2_ and PaO_2_ levels from arterial blood gas analyses corresponding to the worst oxygenation in the first 24 h of ICU stay were retrieved for analyses. Logistic regression analyses were performed to assess the relationship between hospital mortality and both categorized groups and a spline-based transformation of the continuous values of PaCO_2_ and PaO_2_.

**Results:**

In total, 5,258 cardiac arrest patients admitted to 82 ICUs in the Netherlands were included. In the first 24 h of ICU admission, hypocapnia was encountered in 22 %, and hypercapnia in 35 % of included cases. Hypoxia and hyperoxia were observed in 8 % and 3 % of the patients, respectively. Both PaCO_2_ and PaO_2_ had an independent U-shaped relationship with hospital mortality and after adjustment for confounders, hypocapnia and hypoxia were significant predictors of hospital mortality: OR 1.37 (95 % CI 1.17–1.61) and OR 1.34 (95 % CI 1.08–1.66). A synergistic effect of concurrent derangements of PaCO_2_ and PaO_2_ was not observed (P = 0.75).

**Conclusions:**

The effects of aberrant arterial carbon dioxide and arterial oxygen concentrations were independently but not synergistically associated with hospital mortality after cardiac arrest.

**Electronic supplementary material:**

The online version of this article (doi:10.1186/s13054-015-1067-6) contains supplementary material, which is available to authorized users.

## Introduction

Even after successful resuscitation and return of spontaneous circulation (ROSC), cardiac arrest carries a poor prognosis with limited options for treatment [1, 2]. In addition to controlling temperature after cardiac arrest, optimizing ventilation and oxygenation may improve outcome [3]. International consensus currently recommends careful monitoring of post-resuscitation ventilation for neurological and cardiovascular outcome [4]. Indeed, targeting safe levels of carbon dioxide and oxygen in arterial blood may limit global ischemic damage and enhance oxygenation and blood flow. Aberrant arterial levels have repeatedly been shown to be associated with worse outcome after cardiac arrest, but the effects may depend on degree and duration of the (concurrent) exposure [5–14]. Recently, a large cohort study was performed in 125 intensive care units (ICUs) in Australia and New Zealand, which showed that abnormal concentrations of arterial carbon dioxide (PaCO_2_) were common after cardiac arrest [15]. Compared with normocapnia, hypocapnia was independently associated with worse clinical outcomes, whereas hypercapnia was associated with a greater likelihood of good outcome. The results were reproduced in a smaller cohort [16] and are supported by pediatric [17] and experimental research [18–20]. However, ventilation and oxygenation are closely related and effects of PaCO_2_ may not be independent from arterial oxygen levels (PaO_2_). In this study, we aimed to investigate the separate and combined effects of both parameters in a multicenter cohort of patients admitted to Dutch ICUs after cardiac arrest.

## Methods

### Data collection

Analyses were performed on patient data retrieved from 82 ICUs of teaching and non-teaching hospitals participating in the Dutch National Intensive Care Evaluation (NICE) registry between 2007 and 2012. The NICE registry is a high quality ICU database, which is subject to multiple quality checks and local audits in accordance with applicable research and ethical protocols [21]. In brief, the registry contains all clinical data required to calculate mortality risk predictions according to, among others, the Acute Physiology and Chronic Health Evaluation (APACHE) IV for all consecutive ICU patients. The registry does not contain variables determining the cause and circumstances of the cardiac arrest and resuscitation. For the analyses, data obtained from routine care and without patient identifying information were used and consent was therefore not needed according to the Dutch Personal Data Protection Act.

In 2012, approximately 90 % of all Dutch ICUs recorded the data for their patients in the registry. In accordance with the previously conducted study by Schneider et al. [15], all adult patients admitted after out-of-hospital cardiac arrest were included. Abstracted data included demographics, comorbidities, arterial blood gas parameters, diagnostic and physiologic information, admission source and illness severity score by means of the APACHE IV.

### Data extraction

Adult patients admitted to the ICU after out-of-hospital cardiac arrest and cardiopulmonary resuscitation, who were mechanically ventilated at any moment in the first 24 h of admission, were included. We excluded readmissions, trauma patients, nonventilated patients and records not meeting APACHE IV criteria.

As part of the NICE data collection, arterial blood gas (ABG) parameters that were associated with the lowest PaO_2_ to FiO_2_ ratio in the first 24 h after admission were automatically extracted and subsequently used for classification of patients. The APACHE IV score was recalculated (AP4-adj) by standardizing the PaCO_2_ and PaO_2_ to fixed normal values (40 mmHg and 80 mmHg, respectively) in order to prevent overadjustment of these variables in the multivariate models.

### Statistical analysis

Univariate and multivariate logistic generalized estimating equation (GEE) regression models, which account for potential correlation of outcome within ICUs, were used to examine the relationship between the primary outcome (hospital mortality) and either PaCO_2_ or PaO_2_. The relationship of PaCO_2_ and PaO_2_ with mortality was plotted in order to inspect the dose–response curve. Considering the nonlinear relationships, the associations were analyzed by modeling each of PaCO_2_ and PaO_2_ as a restricted cubic spline and separately in categorized groups [22]. PaCO_2_ was categorized in three groups, using conventional thresholds (normocapnia: 35–45 mmHg). PaO_2_ was categorized according to thresholds from previous studies (normoxia: 60–300 mmHg) [5, 7–9, 23, 24]. The individual, joint and interaction effects of two-sided derangements were separately investigated, as suggested for cohort studies [25]. For a further understanding of the dose–response relationship in multivariate models, PaO_2_ categories were also reanalyzed with alternative thresholds derived from observation percentiles or previously used targets [5, 7, 8, 26]. Variables extracted from the first 24 h of admission were considered for the multivariate etiological model based on clinical relevance and in accordance with a previously used model [15]. Considered covariates were introduced separately to the univariate models in order to estimate the unadjusted effect and included age, gender, AP4-adj, year of admission, admission source, therapeutic hypothermia and lowest glucose as a possible proxy-marker of less attentive care [24]. Covariates were subsequently identified as confounders for the outcome using the 10 % change-in-estimate method [27]. Hence, the final multivariate GEE models consisted of age, lowest glucose, AP4-adj and either PaCO_2_ or PaO_2_. Collinearity among the covariates was inspected by estimating Pearson or Spearman correlation coefficients as appropriate. Routine temperature correction of arterial blood gas results is uncommon in Dutch ICUs and was performed according to the participating site’s practice. To account for multiple testing, the statistical significance level for the P-value was set at 0.01.

All analyses were conducted using SPSS version 21 (IBM Corp, Armonk, NY, USA) and R version 3.0.1 (R Foundation for Statistical Computing, Vienna, Austria).

## Results

Data from 6,496 out-of-hospital cardiac arrest patients and 82 hospitals were extracted from the NICE registry and screened for enrollment (Additional file 1: Figure S1). The main reasons for exclusion were no mechanical ventilation (n = 196), missing valid ABG data (n = 379) and not fulfilling APACHE IV criteria (n = 314).

Descriptive characteristics of the 5,258 included patients are summarized in Table 1. The median age was 66 (interquartile range [IQR] 56–76) and patients were predominantly male (69.6 %). Median PaCO_2_ was 42 mmHg (IQR 36–49) and median PaO_2_ was 92 mmHg (IQR 75–124). Of all patients, 21.6 % were classified as hypocapnic, 43.5 % as normocapnic and 34.9 % as hypercapnic. Patients were further classified as hypoxic (8 %), normoxic (89.3 %) or hyperoxic (2.7 %). The majority of patients (87.4 %) were admitted to the ICU from the emergency room of the same hospital. The unadjusted mean APACHE IV score was 117.3, with the normocapnia and the normoxia groups showing the lowest mean (P < 0.001). Groups were relatively balanced in terms of admission source, comorbidities, temperature, glucose and non-respiratory markers.

**Table 1:** Descriptive characteristics

**Characteristic**	All patients	PaCO_2_ group				PaO_2_ group			
		Hypocapnia	Normocapnia	Hypercapnia	*P* value	Hypoxia	Normoxia	Hyperoxia	*P* value
No. (%) of patients	5258	1136 (21.6)	2288 (43.5)	1834 (34.9)		418 (8.0)	4696 (89.3)	144 (2.7)	
**Baseline characteristics**									
Age (years)	66 (56–76)	68 (56–77)	66 (56–76)	65 (55–74)	<0.001	69 (57–78)	66 (56–75)	67 (56–77)	0.02
Male gender, n (%)	3661 (69.6)	737 (64.9)	1601 (70.0)	1323 (72.1)	<0.001	294 (70.3)	3269 (69.6)	98 (68.1)	0.87
Admission source, n (%)									
Operating room from emergency room same hospital	182 (3.5)	32 (2.8)	95 (4.2)	55 (3.0)	0.05	14 (3.3)	166 (3.5)	2 (1.4)	0.38
Emergency room same hospital	4578 (87.1)	981 (86.4)	1972 (86.2)	1625 (88.6)	0.05	367 (87.8)	4077 (86.8)	134 (93.1)	0.08
Operating room from emergency room other hospital	4 (0.1)	1 (0.1)	1 (<0.1)	2 (0.1)	0.74	0	4 (0.1)	0	0.79
Emergency room other hospital	183 (3.5)	41 (3.6)	81 (3.5)	61 (3.3)	0.90	16 (3.8)	166 (3.5)	1 (0.7)	0.17
Home	311 (5.9)	81 (7.1)	139 (6.1)	91 (5.0)	0.05	21 (5.0)	283 (6.0)	7 (4.9)	0.61
Acute renal failure, n (%)	660 (12.6)	154 (13.6)	242 (10.6)	264 (14.4)	<0.001	79 (18.9)	565 (12.0)	16 (11.1)	<0.001
Chronic co-morbidities, n (%)									
Cardiovascular disease	380 (7.2)	94 (8.3)	158 (6.9)	128 (7.0)	0.30	30 (7.2)	340 (7.2)	10 (6.9)	0.99
Renal disease	319 (6.1)	87 (7.7)	137 (6.0)	95 (5.2)	0.04	24 (5.7)	287 (6.1)	8 (5.6)	0.91
Respiratory disease	225 (4.3)	33 (2.9)	72 (3.1)	120 (6.5)	<0.001	26 (6.2)	191 (4.1)	8 (5.6)	0.08
Cirrhosis	44 (0.8)	16 (1.4)	14 (0.6)	14 (0.8)	0.05	9 (2.2)	35 (0.7)	0	<0.01
Cancer	106 (2.0)	25 (2.2)	48 (2.1)	33 (1.8)	0.62	6 (1.4)	96 (2.0)	4 (2.8)	0.57
Markers of severity,									
APACHE IV score	117.3 (29.69)	117.5 (29.10)	114.6 (30.52)	120.4 (28.68)	<0.001	132.2 (29.22)	115.9 (29.47)	119.5 (26.21)	<0.001
APACHE IV risk of death	80.7 (65.3-90.0)	81.2 (66.2-90.2)	79.2 (63.0-89.2)	82.5 (68.2-91.1)	<0.001	88.8 (78.1-94.0)	79.8 (64.3-89.4)	81.9 (64.9-89.4)	<0.001
Physiological parameters obtained within the first 24 h in the intensive care unit									
Temperature									
Highest temperature (°C)	35.7 (34.8-36.9)	35.8 (34.7-37.0)	35.7 (34.8-36.8)	35.8 (34.8-36.9)	0.37	35.7 (34.6-37.1)	35.7 (34.8-36.9)	35.9 (34.9-36.8)	0.88
Lowest temperature (°C)	32.5 (31.8-33.2)	32.4 (31.8-33.4)	32.5 (31.9-33.2)	32.5 (31.9-33.3)	0.55	32.5 (31.8-33.6)	32.5 (31.8-33.2)	32.4 (31.7-33.3)	0.78
Lowest temperature below 34 °C, n (%)	4229 (80.4)	888 (78.2)	1863 (81.4)	1478 (80.6)	0.08	326 (78.0)	3788 (80.7)	115 (79.9)	0.41
Heart Rate									
Highest heart rate, beats/min	103 (87–120)	102 (85–120)	101 (86–119)	105 (90–122)	<0.001	110 (91–128)	102 (87–120)	105 (88–125)	<0.001
Lowest heart rate, beats/min	55 (45–68)	55 (45–69)	53 (44–65)	55 (45–70)	<0.001	55 (45–74)	55 (45–67)	51 (42–67)	0.11
Blood pressure (BP)									
Highest systolic BP (mmHg)	150 (134–171)	150 (134–170)	150 (134–172)	150 (133–170)	0.61	145 (128–165)	150 (134–172)	156 (139–178)	<0.001
Lowest systolic BP (mmHg)	80 (70–90)	80 (71–91)	81 (70–90)	80 (69–89)	<0.001	76 (64–86)	80 (70–90)	82 (70–91)	<0.001
Respiratory rate (RR)									
Highest RR, breaths (min)	23 (20–28)	23 (19–29)	23 (19–28)	24 (20–29)	<0.001	25 (20–30)	23 (20–28)	23 (19–28)	<0.01
Lowest RR, breaths (min)	14 (12–16)	14 (12–16)	14 (11–16)	14 (12–17)	0.03	15 (12–18)	14 (12–16)	14 (12–16)	<0.001
Oxygenation									
PaO_2_ (mmHg)	92 (75–124)	99 (78–136)	94 (76–125)	87 (70–116)	<0.001	51 (44–56)	94 (78–124)	359 (320–438)	<0.001
FiO_2_ (%)	50 (40–70)	45 (40–60)	50 (40–62)	60 (44–90)	<0.001	66 (50–100)	50 (40–70)	98 (67–100)	<0.001
PaO_2_/FiO_2_ ratio	191 (124–272)	227 (157–316)	203 (134–283)	158 (100–228)	<0.001	71 (55–100)	198 (136–272)	440 (361–550)	<0.001
Carbon dioxide									
PaCO_2_ (mmHg)	42 (36–49)	31 (28–33)	40 (38–43)	52 (48–59)	<0.001	45 (38–54)	41 (35–48)	40 (34–48)	<0.001
Metabolic									
Lowest glucose (mmol l^−1^)	6.0 (4.8-7.4)	5.9 (4.8-7.3)	6.0 (4.8-7.3)	6.1 (4.8-7.5)	0.65	6.2 (4.8-7.9)	6.0 (4.8-7.3)	6.1 (5.0-7.8)	0.13
Acid–base balance									
Lowest pH	7.28 (0.12)	7.37 (0.11)	7.30 (0.10)	7.20 (0.12)	<0.001	7.24 (0.14)	7.29 (0.12)	7.26 (0.14)	<0.001
Highest HCO_3_ - (mmol l^−1^)	22.3 (3.92)	20.8 (3.67)	22.1 (3.54)	23.5 (4.16)	<0.001	22.8 (4.70)	22.3 (3.85)	22.0 (3.89)	0.43
Lowest HCO_3_ - (mmol l^−1^)	17.4 (4.33)	16.0 (4.17)	17.4 (4.03)	18.3 (4.54)	<0.001	16.6 (4.96)	17.5 (4.27)	17.2 (4.10)	<0.01

Data presented as total number (percentage), mean (standard deviation) or median (interquartile range) depending on underlying data distribution

P-values for group comparisons using ANOVA or Kruskal-Wallis according to data distribution

*APACHE* Acute Physiology and Chronic Health Evaluation, *ANOVA* Analysis of variance

### Unadjusted outcome

Table 2 shows the unadjusted mortality rates. Overall, 2,491 (47.4 %) patients died in the ICU and 2,833 (53.9 %) died in the hospital. Hospital mortality was highest in the hypocapnia group (58.4 %), compared with the hypercapnia (56.8 %) and normocapnia (49.3 %) group (P < 0.001). Compared with the hyperoxia (57.6 %) and normoxia (52.9 %) groups, hospital mortality was higher (P < 0.001) in the hypoxia group (63.6 %).

**Table 2:** Unadjusted mortality rates

**Outcome**	All patients	PaCO_2_ group				PaO_2_ group			
		Hypocapnia	Normocapnia	Hypercapnia	*P* value	Hypoxia	Normoxia	Hyperoxia	*P* value
Intensive care unit mortality	2491 (47.4)	576 (50.7)	976 (42.7)	939 (51.2)	<0.001	244 (58.4)	2171 (46.2)	76 (52.8)	<0.001
In-hospital mortality	2833 (53.9)	663 (58.4)	1129 (49.3)	1041 (56.8)	<0.001	266 (63.6)	2484 (52.9)	83 (57.6)	<0.001

Data presented as total number (percentage) per group. P-values for group comparisons using Chi-squared test

In the univariate logistic regression model, PaCO_2_ was significantly associated with mortality (P < 0.001). This model was improved when PaO_2_ was added (P < 0.001). No interaction effect (arterial oxygen by arterial carbon dioxide concentration) on mortality was found (P = 0.25). PaO_2_ was also univariately associated with hospital mortality (P < 0.001).

### Adjusted outcomes

**Fig. 1:**
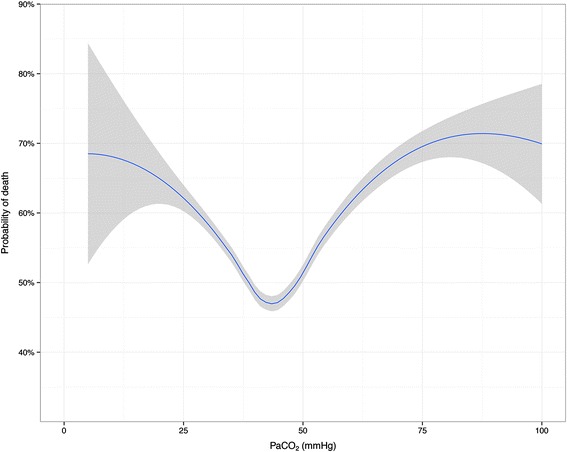
Adjusted probability of in-hospital death by arterial carbon dioxide levels. Loess smoothing curve predicted from logistic regression model adjusted for spline functions of age, lowest glucose, AP4-adj and PaO_2_. Grey zones represent 95 % confidence intervals

**Fig. 2:**
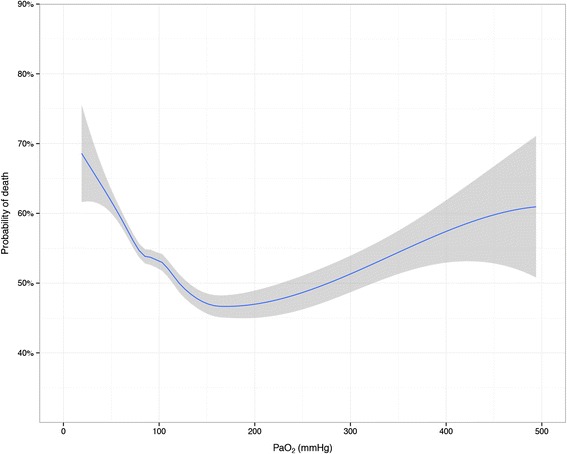
Adjusted probability of in-hospital death by arterial oxygen levels. Loess smoothing curve predicted from logistic regression model adjusted for spline functions of age, lowest glucose, AP4-adj and PaCO_2_. Grey zones represent 95 % confidence intervals

Both PaCO_2_ and PaO_2_ showed a curvilinear U-shaped relationship with mortality in adjusted analyses (Figs. 1 and 2). Odds ratios from multivariate analyses are listed in Table 3. After adjustment for age, lowest glucose, AP4-adj and PaO_2_ (splines), hypocapnia showed a significant association with hospital mortality (P < 0.001), whereas hypercapnia did not. When this model was reanalyzed without adjustment for PaO_2_, the results were virtually unchanged (data not shown).

**Table 3:** Adjusted associations between subgroups and hospital mortality

**Group comparison**	Odds ratio (95 % CI)	*P* value
PaCO_2_ groups		
Hypocapnia vs. normocapnia	1.39 (1.18–1.63)^a^	<0.001
Hypercapnia vs. normocapnia	1.10 (0.95–1.27)^a^	0.20
Hypercapnia vs. hypocapnia	0.79 (0.67–0.94)^a^	<0.01
PaO_2_ groups		
Hypoxia vs. normoxia	1.34 (1.08–1.66)^b^	<0.01
Hyperoxia vs. normoxia	1.13 (0.81–1.57)^b^	0.46
Hyperoxia vs. hypoxia	0.85 (0.58–1.24)^b^	0.39
Alternative PaO_2_ categories^c^		
55-80 vs. >300 mmHg	1.06 (0.76–1.50)^b^	0.72
80-102 vs. >300 mmHg	0.90 (0.64–1.27)^b^	0.55
102-300 vs. >300 mmHg	0.79 (0.56–1.11)^b^	0.17

Hypocapnia = PaCO_2_ < 35 mmHg; normocapnia = PaCO_2_ 35–45 mmHg; hypercapnia = PaCO_2_ > 45 mmHg

Hypoxia = PaO_2_ < 60 mmHg; normoxia = PaO_2_ 60–300 mmHg; hyperoxia = PaO_2_ > 300 mmHg

^a^Multivariable analysis adjusted for age, lowest glucose, AP4-adj and PaO_2_ (splines)

^b^Multivariable analysis adjusted for age, lowest glucose, AP4-adj and PaCO_2_ (splines)

^c^Stratification based on thresholds from ARDSnet oxygenation target (55–80 mmHg), upper threshold of median cohort quintile (102 mmHg), and threshold from previous studies (300 mmHg)

Adjusted for age, lowest glucose, AP4-adj and PaCO_2_(splines), hypoxia but not hyperoxia was found to be associated with hospital mortality in comparison to normoxia (P < 0.01). When this model was reanalyzed without adjustment for PaCO_2_ the results were not materially different (data not shown). When the model was reanalyzed with hyperoxia (>300 mmHg) as reference category, no effects on mortality were observed for various oxygenation ranges.

The individual and joint effect estimates for derangements (normal range vs. outside normal range) of both parameters are listed in Table 4. Aberrant levels of both PaCO_2_ and PaO_2_ were independently associated with hospital mortality (P < 0.01). The estimate for the interaction term (presence of PaCO_2_ derangement by presence of PaO_2_ derangement) was not significant on a multiplicative scale (P = 0.75).

**Table 4:** Associations between derangements and hospital mortality

Variable	Unadjusted odds ratio (95 % CI)	Adjusted odds ratio (95 % CI)	*P* value
PaCO_2_ derangement vs. normocapnia	1.38 (1.24–1.54)	1.21 (1.07–1.36)^a^	0.003
PaO_2_ derangement vs. normoxia	1.45 (1.21–1.74)	1.27 (1.05–1.54)^b^	0.01
Interaction term	-	1.07 (0.71–1.62)	0.75

PaCO_2_ derangement = PaCO_2_ < 35 or PaCO_2_ > 45 mmHg; normocapnia = PaCO_2_ 35–45 mmHg

PaO_2_ derangement = PaO_2_ < 60 or PaO_2_ > 300 mmHg; normoxia = PaO_2_ 60–300 mmHg

^a^Multivariable analysis adjusted for age, lowest glucose, AP4-adj and PaO_2_ (splines)

^b^Multivariable analysis adjusted for age, lowest glucose, AP4-adj and PaCO_2_ (splines)

*CI* confidence interval

## Discussion

In accordance with previous studies, we found that early exposure to both hypo- and hypercapnia is common in ICU patients resuscitated from cardiac arrest [15, 16]. In contrast, hypoxia and severe hyperoxia are uncommon findings early in the ICU stay of Dutch hospitals. Both PaCO_2_ and PaO_2_ had a U-shaped relationship with outcome and after adjustment for known confounders, hypocapnia and hypoxia were significantly associated with hospital mortality. Hyperoxia was not independently associated with higher mortality in comparison with various ranges for normoxia. However, this study may lack power to detect significant associations for severe arterial oxygen derangements considering the low prevalence in the present cohort.

Our adjusted mortality plots and the categorized results stress the importance of aberrant arterial levels after cardiac arrest, but rigid cut-offs for optimal ranges remain to be determined and validated. Increasing mortality rates may be skewed towards extreme PaO_2_ levels in the early phase after cardiac arrest. In line, PaCO_2_ levels between 40 and 45 mmHg appear to be favorable shortly after ICU admission. The complex U-shape of the survival curves for both parameters may explain the heterogeneity in previously observed associations [14]. It shows that unfavorable effects cannot be consistently captured when the results are stratified by groups based on arbitrary thresholds. Indeed, studies assessing arterial hyperoxia with lower thresholds usually failed to show significant effects on outcome, whereas higher risks were observed with substantially higher upper limits [5–9]. The current findings validate the recent calls for caution with hyperoxia in cardiac arrest patients only to a limited extent. The prevalence in this cohort shows that hypoxia and hyperoxia are not a common concern shortly after cardiac arrest patients are admitted to Dutch ICUs. In the analyses of those conditions, the relatively small number of exposed patients increases the probability of type 2 errors. Associations are, therefore, more likely to be consistent with increasing statistical power in the studied subgroups. Moreover, reanalyzing the adjusted effects of oxygenation based on quintiles did not detect a significant association with mortality (data not shown). Hypothesizing that physicians would avoid hypoxia most attentively in the most critically ill patients, hyperoxia could be an indirect marker of illness severity or responsive care, and could thereby reflect a worse outcome. Accordingly, hypoxia and hypocapnia may also be markers of less attentive care or prehospital injury.

The absence of a significant interaction effect between PaCO_2_ and PaO_2_ suggests that it is mainly the effect of the individual variables that influences mortality in our model rather than the absolute effect caused by the interaction between the two variables. The effect of PaO_2_ on hospital mortality is therefore not likely to differ significantly across strata of PaCO_2_, or vice versa. Further, the effect size did not significantly depend on the concurrent presence of aberrant arterial carbon dioxide and arterial oxygen levels. Conditions in which both parameters are concurrently and strongly modified may, therefore, not synergistically increase the risk. However, the univariate associations of PaCO_2_ and PaO_2_ were subtly altered when adjusted for each other and both parameters should, therefore, judiciously be considered as possible confounders.

For our analyses, we were restricted to the variables that were collected as part of the NICE registry. Our database does not contain prehospital variables, nor does it include all ABG samples per admission, but only a single measurement associated with the worst oxygenation in the first 24 h. Although this method has not previously been shown to be inferior, the selected data may not be the most representative data over the total ICU stay and may, therefore, misclassify patients. In addition, selecting either the first, worst or highest value from arterial blood gas sampling emerges as an essential methodological issue for the intended analyses [28]. The first measured sample may reflect pre-ICU treatment, including oxygen administration in the ambulance and emergency department. Early oxygen administration can influence oxidative metabolism, respiratory markers, vasoconstrictive status and blood flow [29–31], and may thus be an important predictor of outcome. In fact, both highest and lowest systolic blood pressures were significantly higher in the hyperoxia groups. Further, hyperoxia frequently coincides with hyperventilation and concurrent hypocapnia [32]. Interestingly, systemic blood pressures were very similar across the PaCO_2_ subgroups in this cohort. PaCO_2_ could yet be an important mediator in vascular effects, cardiopulmonary resuscitation and cerebral perfusion [13, 33]. In view of that, the association between hypocapnia and mortality may be explained by cerebral vasoconstriction, whereas hypercapnia may be less harmful due to increased peripheral tissue oxygenation [34–38].

Although our findings are observational and do not necessarily imply causality, the present results are supported by previous results [5, 15]. Our findings regarding hyperoxia are in line with several recent studies [8, 23, 39], even though conflicting results have been documented [6, 7, 24, 40]. Parts of the heterogeneity in previous findings may be attributed to the adjustment for PaCO_2_. Pure oxygen therapy after cardiac arrest has previously been shown to worsen neurological outcome in animal models [41] and exposure to hypocapnia and hypercapnia after ROSC has been associated with poor neurological function at hospital discharge [16]. However, the effects of PaO_2_ targets on neurological recovery of critically ill patients are still uncertain.

In contrast to the previous study by Schneider et al. [15], both the unadjusted and adjusted association between mortality and hypocapnia were statistically significant. Specific study differences may be explained by population and methodological differences. Our multivariate model differed slightly and there was less dispersion of the carbon dioxide concentrations in our data. Other notable differences between both studies include the substantially lower median PaO_2_ (92 vs. 106 mmHg), mean FiO_2_ (58 vs. 71 %), and marginally lower mean PaCO_2_ (46 vs. 44 mmHg). Furthermore, the vast majority of patients in our cohort (80 vs. 40 %) reached a temperature lower than 34 °C during the first 24 h of ICU admission. Under these conditions, PaCO_2_ and PaO_2_ progressively decrease with decreasing body temperature and the occurrence of hypocapnia and hypoxia may be underestimated with uncorrected ABG levels. However, temperature correction of ABG measurements is ambiguous and was not routinely performed in our study or in the study by Schneider et al.

In order to consistently assess the relationship between risk factors and outcome, it is important to re-evaluate previously established associations in different populations using robust methodology. The modified methodology of the present study provides further insights into the independent and combined effects of PaCO_2_ and PaO_2_ and accounts for clustering by hospital, interaction effects and model variances. Still, residual confounding by prehospital and Utstein variables cannot be ruled out, and derangements may not be isolated risk factors for mortality.

## Conclusions

In this multicenter cohort study, we have studied the survival probability inferred from different levels of PaCO_2_ and PaO_2_ in post cardiac arrest patients. Most effects were attenuated after adjustment for identified confounders, but hypocapnia and hypoxia were independently associated with hospital mortality. The close relationship between both parameters argues for a concurrent assessment of the effects and further evaluation of target ranges is warranted.

## Key messages

- After resuscitation from cardiac arrest, exposure of patients to both hypo- and hypercapnia is common within 24 h of ICU admission
- Hypoxia and severe hyperoxia are uncommon findings early in the ICU stay
- Both PaCO_2_ and PaO_2_ had an independent U-shaped relationship with hospital mortality
- After adjustment for relevant confounders, hypocapnia and hypoxia were significant predictors of hospital mortality
- A synergistic effect of concurrent derangements of PaCO_2_ and PaO_2_ was not observed

## Additional file

Additional file 1: Figure S1.
**Supplementary file could not be accessed online for author review**. (PDF 193 kb)

## Abbreviations

ABG: Arterial blood gas
APACHE: Acute physiology and chronic health evaluation
GEE: Generalized estimating equation
ICU: Intensive care unit
NICE: National intensive care evaluation (Dutch ICU registry)
PaCO_2_: Arterial concentrations of carbon dioxide
PaO_2_: Arterial concentrations of oxygen
ROSC: Return of spontaneous circulation
