# Supplementary figures and images for: Associations of arterial carbon dioxide and arterial oxygen concentrations with hospital mortality after resuscitation from cardiac arrest
Source: Crit Care. 2015 Sep 29;19:348. doi: 10.1186/s13054-015-1067-6 (PMC4587673; doi:10.1186/s13054-015-1067-6)

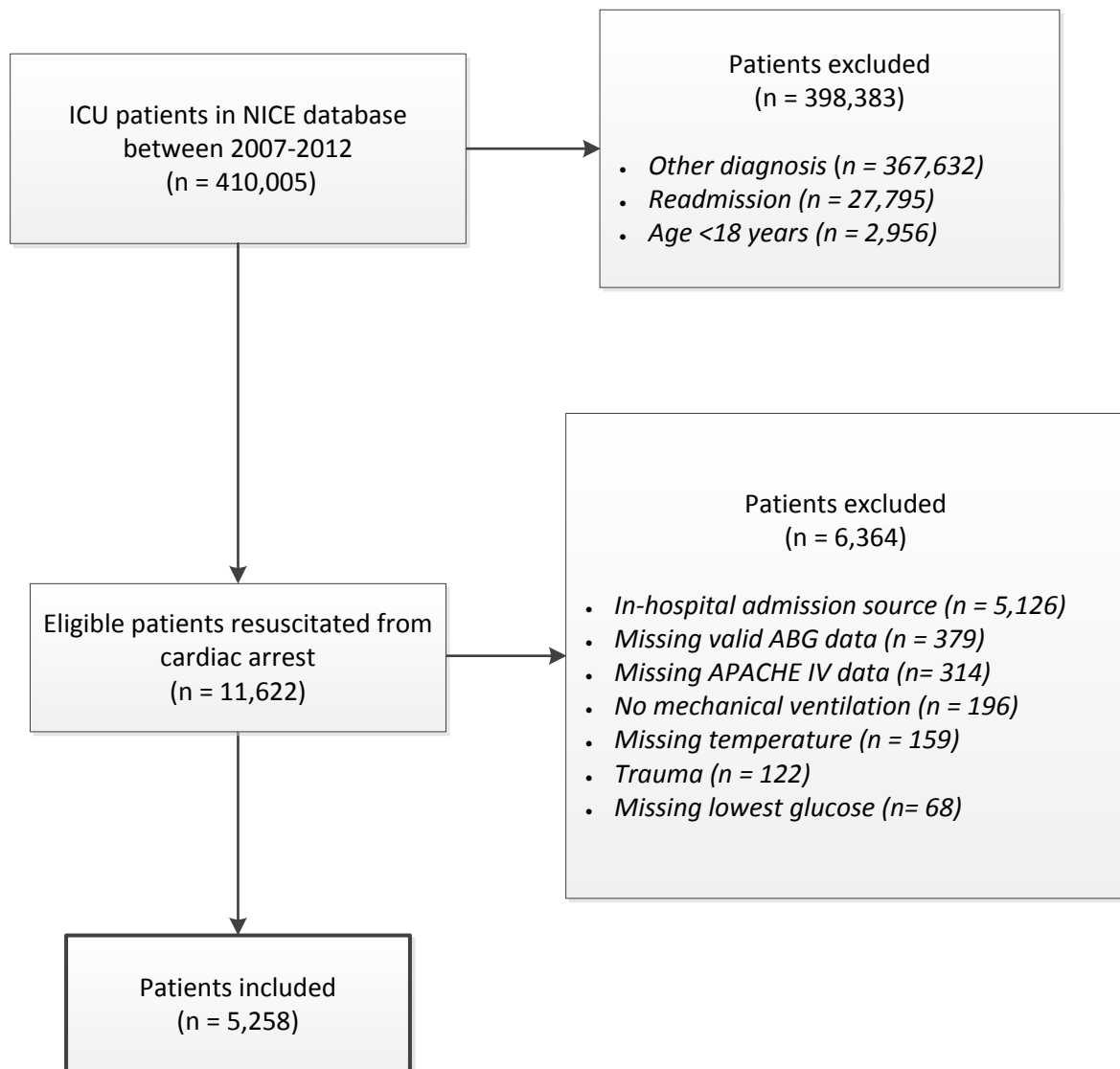

Supplement: Additional file 1: Figure S1. — Supplementary file could not be accessed online for author review. (PDF 193 kb) [file 13054_2015_1067_MOESM1_ESM.pdf]
